# The Predictive Role of Chinese English as a Foreign Language Teachers’ Psychological Capital in Their Job Commitment and Academic Optimism

**DOI:** 10.3389/fpsyg.2022.916433

**Published:** 2022-07-18

**Authors:** Lihua Xu, Xiaowen Zhu

**Affiliations:** ^1^School of Humanities and Social Science, Xi’an Jiaotong University, Xi’an, China; ^2^School of Humanities and Foreign Languages, Xi’an University of Post and Telecommunications, Xi’an, China

**Keywords:** positive psychology, psychological capital, job commitment, academic optimism, language learning, EFL teachers

## Abstract

Positive psychology (PP) has received increasing attention in the field of education. Therefore, it would be of utmost importance to analyze the relationship between the constructs falling under the category of positive psychology and other constructs to pave the way for both educational authorities and teachers themselves. Reviewing the previous studies, it turned out that there have been no studies to discuss the interplay between teachers’ psychological capital and their job commitment and academic optimism particularly in the context of China. To fill this gap, the current study set out to inspect the function of Chinese English as a Foreign language (EFL) teachers’ psychological capital in promoting their job commitment and academic optimism. To accomplish this, 316 Chinese teachers (i.e., 97 males, 219 females) were asked to complete “Psychological Capital Scale,” “Job Commitment Scale,” and “Academic Optimism Scale.” The results of regression analyses revealed that Chinese EFL teachers’ psychological capital can strongly predict their job commitment and academic optimism. The conclusion and implications of the results are finally discussed and it is mentioned that both educational authorities and teachers can benefit from this study and make the experience of teaching much more productive.

## Introduction

Teaching has been perceived to be a stressful and demanding career, with several psychological issues associated with it (Montgomery and Rupp, 2005). When teachers feel stressed-out to any degree while working and when they do not feel psychologically well, students’ academic and behavioral outcomes will be negatively and tremendously affected (Wentzel et al., 2010). Attention has been drawn to teachers’ psychological capital recently (Collie et al., 2012) and the number of these studies is on the rise since if teachers feel livid, it affects their commitment and optimism, leading to some mental problems (Sutton and Wheatley, 2003). Teachers’ burnout, which is rooted in not being actively involved in work, has a negative impact not only on teachers themselves, but also on students because teachers cannot stand students’ behavioral problems or problems that can be found in the learning process (Ross et al., 1989), contributing to not having a good relationship with those students, being absolutely strict and not feeling committed and indulged in their job (Friedman, 2004). In spite of the fact that teachers’ crucial role has attracted consideration and has been viewed as a radical factor in second language acquisition (MacIntyre et al., 2016), more studies should be carried out in this field, psychological capital to place emphasis on the important role of teachers’ well-being in both teachers’ commitment and academic optimism (AO). This study, thus, is of crucial importance as it evaluates the relationship between two significant factors. First of all, to the researchers’ best knowledge, there have not yet been any studies discussing the association between TPC and their job commitment (JC) and AO. Secondly, such studies with the mentioned variables have not been carried out in China. Last but not least, many variables can be investigated but what is important is the link between these two variables which can help the authorities strengthen the education system, regarding the variables. Accordingly, the current study aims to answer the following question to pave the way for future studies in this field.

• Does Chinese English as a Foreign language (EFL) teachers’ psychological capital predict their commitment and academic optimism?

## Literature Review

### Psychological Capital

Psychological capital has attracted attention after positive organizational behavior theory has arisen. The organizational atmosphere causes many problems. Based on Luthans et al. (2007b), psychological capital has four components: hope, optimism, efficacy, and resilience which enables teachers to enhance their well-being. Hope theory was designed by Snyder’s (2002) which puts emphasis on the fact that both successful agency and pathways can be considered as important, considering the theory of hope, meaning that they have enough energy to reach the goals for which they have plans. It has been discovered that those with high hopes can do the tasks better in the following fields: athletics, psychological modifications, psychotherapy, and other areas (Curry et al., 1997; Kwon, 2002; Reichard et al., 2013). Then hope theory was used in the educational domain owing to its unbelievable importance (Gallegher et al., 2017).

Self-efficacy is another subfactor of psychological capital which is conceptualized as one’s beliefs about his abilities to achieve specific accomplishments (Tschannen-Moran et al., 1998). It affects many factors, for example, the way they make effort to reach their goals, how resilient and determined they are when they are faced with a problem, how they can put up with a hard situation such as a failure, and how stressed-out they are when they are put under pressure (Bandura, 1977). Teacher self-efficacy is of great importance because it can tremendously impact schools and students’ lives. With this in mind, teacher self-efficacy is conceptualized as teachers’ beliefs about the degree to which they can influence their students’ efficiency. Therefore, the effort teachers put into teaching, the way the goals are set and how realistic they might be, the amount of passion and ambition they express in their job are all impacted by teachers’ sense of efficacy. Self-efficacious teachers are believed to be more enthusiastic and more responsible about their job and they are highly likely to stay in their job for the rest of their lives (Tschannen-Moran et al., 1998). Regarding that, self-efficacy is one of the significant components for teachers that help many positive behaviors in teaching to be activated, leading to prosperous outcomes for students (Henson et al., 2001; Derakhshan, 2022). Teachers with high self-efficacy are thought to be more persistent when running into problems and coping with less motivated students. Their teaching effectively impacts these students’ development (Gibson and Dembo, 1984). Moreover, they do not resist new experiences and express more commitment (Guskey, 1988; Coladarci, 1992).

The third factor of psychological capital that should be highlighted is resilience which is defined as one’ capability to overcome the bad situation and stress in order to become a happier and stronger person after a complicated situation (Luthans et al., 2007b). Teacher resilience causes balance in various aspects of a teacher’s job (Hiver, 2018; Ergün and Dewaele, 2021; Wang et al., 2021). Teachers’ resilience is the ability to acclimatize to different situations and raise their competence when encountering bad conditions (Gordon and Coscarelli, 1996). According to what has been claimed by Kitching et al. (2009) several items helping to make teachers able to develop rather than just survive in their jobs have been dealt with. Moreover, resilient teachers are perceived to be more committed to their workplace and it is more probable to help their colleagues if they confront problems, to develop a friendly working ambience, to put up with inconveniences without complaining, and to be more pleased with their jobs; therefore, they can manage the classes better (Witt, 1991). It can be taken into account that it is a higher probability for resilient teachers to have control in class, manage the class more effectively, achieve their goals, decide more wisely, and lead a productive and healthy life (Salgado, 2005).

Finally, optimism is another factor of psychological capital. Scheier and Carver (1985) coined this term that is viewed as a positive outlook on life (Seligman, 2002) and it is also thought that positivity or negativity is linked to the way people tackle their problems. It has been stressed in some studies that teachers with high levels of optimism are highly likely to teach more effectively because they can be risk-takers. Moreover, they grow a positive approach to their students and obstacles are regarded as new opportunities that can be dealt with (Duckworth et al., 2009).

### Job Commitment

Commitment can be categorized into three classifications. The affective commitment that is concerned with one’s emotional bound to his organization and the amount to which he is involved in it. These employees are inclined to stay in their jobs. Several mental processes are associated with developing affective commitment, retrospective authenticity, personal fulfillment, and classical conditioning, to name a few (Meyer and Allen, 1997). Based on what has been mentioned in literature there are some situations in which affective commitment can be developed in employees, for instance, to communicate honestly in an organizational structure (Zangaro, 2001), to have a positive participation in an organizational culture (Parnell and Crandall, 2003), when they have the allowance to make strategically significant decisions (Celep, 2000; Somech and Bogler, 2002; Lines, 2004), when the outcomes of these decisions have an impact (Torka, 2004), when they are given autonomy (Firestone and Pennell, 1993), when they are given the opportunity to reach the resources of knowledge and other things in the organization (McDermott et al., 1996), when the strategic aims (Enriquez et al., 2001), anticipations (O’Creevy et al., 1997), and the vision (Oswald et al., 1994) of organization are discussed with them, when they are fairly treated (Martin and Bennett, 1996; Naumann et al., 1998), when they are adequately paid (Abdulla and Shaw, 1999), when there are shared ethical values between the organization and the employees (Schwepker, 1999; Peterson, 2003; Janssen, 2004), when the leader supports the employees and facilitate the process for them (Kidd and Smewing, 2001; Hui et al., 2004), and when the leader can be trusted by the employees (Perry, 2004).

Continuance commitment is concerned with the degree to which an employee is aware of the costs linked with giving up an organization and the advantages relevant to staying in an organization. Such employees are found to stay in an organization (Meyer and Allen, 1997). Because teaching takes time and effort, employees might think that if they want to leave their workplace, they will be forfeited. Another reason for continuance commitment is to what extent an employee feel assured to be able to have the employment alternatives. In other words, the more certain they are that they can access the employment alternatives, the less likely they are to leave their job, and the stronger the continuance commitment would be (Meyer and Allen, 1997). Many employees who are getting older find it difficult to leave their organization due highly to the costs related to leaving since they have already invested much time and energy and they all think about their positions, retirement money, unused vacations, job security, etc. (Allen and Meyer, 1993; Abdulla and Shaw, 1999).

Normative commitment is concerned with a sense of being obliged to pursue employment. These employees are more likely to stay with an organization for long. This commitment is rooted in the pressures that one feels due to his values which are relevant to their family, culture, and organization. These values can be learned through conditioning (giving rewards and reprimands) and modeling (observing and imitating others) (Meyer and Allen, 1997).

### Academic Optimism

Academic optimism can be defined as a teacher’s optimistic belief that he comes to the recognition that differences can be made in students’ academic performance. Such teachers believe in their capabilities to address the problems and failures are regarded as stepping stones that take resilience and diligence to be resolved. Academic optimism can be classified into three categories, teachers’ self-efficacy, teachers’ trust in learners and parents, and teachers’ sense of concentration on providing a positive controversial environment for education for students. Teachers’ sense of efficacy is defined as the amount to which teachers judge their capabilities to give rise to pleasant outcomes of how students are engaged in the class and learn, considering the students who are demotivated and problematic (Tschannen-Moran et al., 1998). This feature in teachers is associated with students’ academic achievements. The reason behind it is if teachers do believe in themselves and the way their students’ success and achievements can be affected by them, higher expectations are set by them, and they make greater effort to make it done, and as a result they overcome the challenging moments with which they are faced. Moreover, trust should be built between teachers and students and also their parents which causes a person to be benevolent, reliable, honest, and open (Hoy and Tschannen-Moran, 2003). This trusting relationship allows both sides to be open and honest toward each other, leading to their betterment. It also forms a sense of reliability, causing students to rely on their teachers and their teaching methods and assuring teachers to trust their students’ effort to practice what they have been taught. Additionally, teachers’ sense of benevolence helps students to trust in their teachers since they want their students to be successful and achieve their goals. Optimism was first examined by Seligman (1998) who is a positive psychologist. To him, positivism would be as significant as capability and motivation. Optimism is when someone expects something in a positive way for the things that are going to happen (Carver and Scheier, 2002). When one has faith in the future, it creates a positive effect on what he is doing today and it defines how a person should act to achieve his goal (Peterson and Park, 2004).

Optimistic people are believed to always see the glass as half full and it is what causes them to keep hopeful and motivated in the face of difficulties (Schueller and Seligman, 2008). While, educational optimism is relevant to individuals’ tendencies like attitudes toward something and a positive understanding of the future events, academic optimism is just about the teaching and learning contexts. Academic optimism is a phrase that was coined by Hoy et al. (2006) and as it was discussed it is different from educational optimism. Academic self-efficacy, academic significance, and trust fall under the category of academic optimism which is meaningful at the educational and organizational level (Hoy and Tarter, 2011).

As stated, academic self-efficacy is conceptualized as one’ belief in showing behaviors successfully to provide the planned outcomes (Bandura, 1977). In other words, the way teachers assess their abilities to complete an activity (Hoy and Miskel, 2001). Academic significance or emphasis is the degree to which schools are expecting perfection and how much pressure they place to see students’ academic success (Beard et al., 2010). It can be taken as an example that when teachers provide their students with a positive academic ambience in which learners are supposed to complete some tasks and do their homework, it is an emblem of academic emphasis (Hoy et al., 2008). Trust is defined as a significant component resulting in learning in schools. Trust is aligned with the efficiency of schools and the positive environment of schools (Tschannen-Moran and Hoy, 1998).

## Materials and Methods

### Participants

Participants of this study were 316 EFL teachers (male = 97, 30.7%, female = 219, 69.3%) whose age ranged from 27 to 67, with an average of 40.5. With different educational levels and academic qualifications (undergraduate = 13.61%, postgraduate = 88.76%, doctor = 2.37%), they were currently studying or teaching in various institutes, colleges and universities in two municipalities and 14 provinces of China with the majority in Shaanxi province (301, 95.27%). They have gotten different years of teaching experiences; almost half of them turned out to be studying or teaching for 11 to 20 years. Their major involves English, linguistics, Business English, Translation, English Teaching and English Literature, among which English itself accounts for the largest proportion (154, 48.73%). All participants had been given consent before they participated in this study. All the responses they provided were totally based on their willingness.

### Instruments

Psychological Capital Scale was developed to determine how psychological capital is perceived by teachers. The scale comprises 24 items. The scale was prepared using a 5-point Likert type response method from strongly disagree (1) to strongly agree (6). Reliability analysis was done. Cronbach’s alpha revealed that the Teachers’ Psychological Capital questionnaire (*r* = 0.91) had satisfactory reliability indices.

In order to measure teachers’ commitment, the EFL Teacher’s Job Commitment Scale was utilized (Meyer et al., 1990). It comprises 18 items, a five-point scale from strongly disagree (1) to strongly agree (5). Reliability analysis was done. Cronbach’s alpha revealed that the Teacher job commitment questionnaire (*r* = 0.91) had satisfactory reliability indices. To measure EFL Teacher’s Academic Optimism, a scale with the same name developed by Tschannen-Moran and Woolfolk Hoy (2001) was used. The scale was prepared using a 5-point Likert type response method from strongly disagree (1) to strongly agree (5). Reliability analysis was done. Cronbach’s alpha revealed that the Academic Optimism questionnaire (*r* = 0.96) had satisfactory reliability indices.

### Data Collection Procedures

Data was smoothly collected in the middle of March by Wenjuanxing (an online data-collection program which is widely used in China) *via* Wechat or other electronic devices. The whole process lasted for two months from January to March. To make the questionnaire easily and clearly understood, researchers had invited two experts in translation and linguistics to translate the questionnaire from English to Chinese and had examined any mistake in the questionnaire before it was typed into the data-collection platform. In order to enhance the reliability of this study, the questionnaire was distributed to different provinces of China where teachers with various majors and experiences were studying or teaching. All participants were given instructions on how to fill in the questionnaire properly and methods of how to deal with any accident that they may encounter in the process. They were ensured that all collected data would remain confidential and only be used for research purposes. Altogether, 318 questionnaires (valid = 316) were collected. The collected data had been double checked for any potential mistake before they were sent to SPSS software for analysis, which would pave the way for the probe and exploration into the research questions.

## Results

In order to decide on the data analysis, preliminary measurements should be done. The first step is to measure the reliability of the three questionnaires used in this study.

To measure the reliability indices of all three questionnaires, Table 1 shows that the process of calculation was repeated three times and the outputs of Cronbach’s alpha revealed that the Teachers’ Psychological Capital questionnaire (*r* = 0.91), Teacher job commitment questionnaire (*r* = 0.91), and Academic Optimism questionnaire (*r* = 0.95) had satisfactory reliability indices.

**TABLE 1:** Reliability of the questionnaires.

Questionnaires	Cronbach’s alpha	No. of items
Teachers’ psychological capital	0.912	24
Commitment	0.912	18
Academic optimism	0.969	40

One of the ways the researcher used for making decisions about using parametric or non-parametric analysis in a quantitative study, is to measure the normality of the data. Table 2 shows the Kolmogorov-Smirnov index which shows that the distribution of data is normal (sig = 0.200) for two of the variables (Psychological capital and job commitment). The assumption for having a normal set of data is to have a non-significant index of K-S, but the output revealed that the data normality rule is violated for academic optimism in this study.

**TABLE 2:** Tests of normality.

	Kolmogorov-Smirnov^a^			Shapiro-Wilk		
		
	Statistic	df	Sig.	Statistic	df	Sig.
PC	0.043	315	0.200*	0.980	315	0.000
JC	0.040	315	0.200*	0.996	315	0.563
AO	0.097	315	0.000	0.976	315	0.000

*This is a lower bound of the true significance.

*^a^Lilliefors significance correction.*

### The Research Question

Does EFL teachers’ psychological capital predict their commitment and academic optimism?

Since the research question includes one independent and two dependent variables regarding the predictability power, the researcher ran two linear multiple regression analyses. Before doing linear multiple regression analyses, reliability of the questionaire in Table 1 and tests of normality in Table 2 were run to ensure the validity of statistical analysis.

Table 3 provides a model summary for teachers’ psychological capital and job commitment. It was shown that the model, which contains the scores of teachers’ psychological capital, can explain the amount of variance in the dependent variable (job commitment). This model can explain 22.20% of the variances in the teachers’ job commitment.

**TABLE 3:** Model summary for Chinese EFL PC and JC.

Model	R	R square	Adjusted R square
1	0.472^a^	0.222	0.220

*^a^Level of significance.*

Table 4 labeled ANOVA tested the hypothesis that multiple R in the population equals zero (0). The model reached statistical significance [*F*_(1,313)_ = 89.52, Sig = 0.000, this really means *p* < 0.05].

**TABLE 4:** ANOVA for Chinese EFL PC and JC.

Model		Sum of squares	df	Mean square	*F*	Sig.
1	Regression	10013.990	1	10013.99	89.52	0.000^b^
	Residual	35013.197	313	111.86		
	Total	45027.187	314			

*^a^Dependent variable: JC.*

*^b^Predictors: (Constant), PC.*

To measure whether the independent variable (teachers’ psychological capital) can predict the dependent variable (teachers’ job commitment), the sig. column was studied. As shown in Table 5, psychological capital is a significant predictor of teachers’ job commitment (sig = 0.000, *B* = 0.47). To measure the predictability power of Chinese EFL teachers’ psychological capital and academic optimism, a linear multiple regression analysis was run. The results of the analysis were shown in the tables below.

**TABLE 5:** Coefficients for Chinese EFL PC and JC.

Model		Unstandardized coefficients		Standardized coefficients	*t*	Sig.
			
		B	Std. error	β		
1	(Constant)	30.33	4.15		7.31	0.000
	PC	0.380	0.04	0.472	9.46	0.000

*^a^Dependent variable: JC.*

Table 6 provides a model summary for teachers’ psychological capital and academic optimism. It was shown that the model, which contains the scores of teachers’ psychological capital, can explain the amount of variance in the dependent variable (academic optimism). This model can explain 4.20% of the variances in the teachers’ academic optimism.

**TABLE 6:** Model summary for Chinese EFL PC and AO.

Model	R	R square	Adjusted R square	Std. error of the estimate
1	0.204^a^	0.042	0.039	27.20619

*^a^Predictors: (Constant), PC.*

Table 7 labeled ANOVA tested the hypothesis that multiple R in the population equals zero (0). The model reached statistical significance [*F*_(1,313)_ = 13.61, Sig = 0.000, this really means *p* < 0.05].

**TABLE 7:** ANOVA for Chinese EFL PC and AO.

Model		Sum of squares	df	Mean square	*F*	Sig.
1	Regression	10080.01	1	10080.01	13.61	0.000^b^
	Residual	231675.36	313	740.17		
	Total	241755.37	314			

*^a^Dependent variable: AO.*

*^b^Predictors: (Constant), PC.*

To measure whether the independent variable (teachers’ psychological capital) can predict the dependent variable (teachers’ academic optimism), the sig. column was studied. As shown in Table 8, psychological capital is a significant predictor of teachers’ academic optimism (sig = 0.000, *B* = 0.20).

**TABLE 8:** Coefficients for Chinese EFL PC and AO.

Model		Unstandardized coefficients		Standardized coefficients	*t*	Sig.
			
		*B*	Std. error	β		
1	(Constant)	149.830	10.675		14.036	0.000
	PC	−0.381	0.103	−0.204	−3.690	0.000

*^a^Dependent variable: AO.*

## Discussion

This study aimed to find the association between teacher psychological capital, job commitment, and academic optimism. The findings revealed that teacher psychological capital is positively correlated with both commitment and academic optimism in that a teacher will be more committed and have higher levels of academic optimism if their psychological capital is high in them (Luthans et al., 2007a). According to what has been achieved in this study, positive psychology capital features six dimensions: self-efficacy, optimism, confidence, extroversion, psychological resistance, and hope. The results of this study showed a positive correlation between two variables of this research. Self-efficacy as one of the components of teacher psychological capital can be found in many aspects of a teacher’ life. They are confident at all stages of their work, meaning that they believe in what they do and the activities they run throughout the class. This causes them to have faith in what they do and positively motivate their students, and as a result of which students feel extrinsically motivated to learn a new language.

Another component that could be taken into consideration is that they know what they have to do to succeed which means not only are they aware of their goals, but they also know how those goals can be achieved, this *per se* provides teachers with complete belief in themselves, leading them to be actively involved in what they do. Optimism, which is one of the dimensions of psychological capital (PC), can be depicted in some examples. Optimistic teachers are full of life and energy. To them, life is good and they are mostly cheerful because they think living in the society gives them a sense of peace. Being optimistic allows teachers to have a positive approach to their work life and it causes them to see failures as a stepping stone, so they have never been blocked in the face of problems, therefore, it makes them stronger in what they do.

Confidence as another dimension of TPC can be exemplified as follows. Confident teachers are aware of their professional responsibilities. It means that their responsibilities can be shouldered by teachers and what causes them to feel satisfied with their jobs is that they can take their responsibilities even if there would be some problems not allowing them to do their best. Confident teachers are also eager to solve students’ problems. Those teachers who are diffident cannot handle their emotions and even their negative thoughts cannot be regulated, that is the reason why they are always stressed. Hence, this stress does not allow them to tackle their problems or to find a solution to their students’ problems. Accordingly, when students’ difficulties are not addressed, they feel demotivated to enjoy the learning process and teachers themselves do not feel satisfied with their jobs. Teachers with higher confidence think that they are efficient in their profession and that is why they are more likely to feel motivated in what they do. Extraversion as another dimension of TPC can be described as follows: if there is a problem in their workplace, they have the courage and determination to meet the authorities if it seems necessary to resolve the problem. Those who are activists have an inclination to talk about their problems so as to find a solution to them while those who are not, are inclined to complain about the situation with which they are not satisfied.

Another component which is highlighted is that extroverted teachers can develop new ideas for their workplace and their preference goes to transparency in their professional life. Open-minded people are capable of not feeling dogmatic and biased and they have the heart to listen to the ideas that oppose theirs, hence, they can come up with new ideas after they have given various types of ideas enough thoughts. Beyond doubt, teaching is a job for which creativity seems necessary and without which teachers could not have been phenomenally successful. The more creative a teacher is, the more engaged he is in his job. And those teachers who are extroverted are inclined to be honest and transparent with people around them and their students since honesty is the number one priority for this profession, teaching. It can be taken as an example, when learners have difficulty acquiring a language, their teachers could find and address the problem if there were any educational problems; therefore, being honest with them and talking about their problems if there were any seems vitally important and it would be another aspect of being engaged at work.

Another dimension of TPC is psychological resistance. When teachers deal with negative events just for the sake of education, they sacrifice their energy for their students’ education. They also think of solutions to the unforeseen problems just due to the fact that they can resist hardships whenever faced and these problems are not as viewed as blockage, instead they learn from them and it is what makes them more interested in their job and causes them to feel more committed toward their lives. Moreover, these difficulties are perceived as challenges which makes them more willing to resolve the problems and sometimes they also fight against difficulties to achieve a better result. It should be kept in mind that for a teacher to be motivated enough, it would be of great importance not to feel easily annoyed, when encountering difficulties. Work engagement, thus, is highly dependent on teachers’ mindsets and the way they see their problems. Hope as the last dimension of TPC can be expressed through different types of behavior. Hope can be regarded as the fruit of psychological resistance. Hopeful teachers can describe their problems as an experience which makes them mature and helps them rebuild their personalities and sometimes the situation makes it even worse that some problems need to be addressed simultaneously and it is what causes teachers to be ready to face the challenges of their job and to actively work. All the dimensions discussed above delineate that TPC is positively correlated with TWE and that is why the findings of this research are of utmost importance.

With regard to academic optimism, when teachers feel highly confident about their working competence and their academic ability, there is a greater possibility to help students recognize their strengths and weaknesses so as to achieve a better result, contributing to students’ reliability in their commitments. In other words, they can be relied on to consider doing their responsibilities relevant to learning and achieving the goals having been set for them by their teachers. Another essential point which can be taken into account is that giving student’s time to work together has always been a priority in classes with teachers having a great psychological capital. Moreover, students are involved in evaluating their own work and setting their own goals which causes them to feel independent, which is an ultimate goal regarding teaching.

According to Maslows’ hierarchy of needs, the highest level to which human beings are supposed to reach is self-actualization and there is a meaningful link between students’ feeling autonomous and actualized, leading to having a fulfilled life. Therefore, learning is a miniature version of real life in which we are expected to be decisive, be committed to what we do, and find our talents. Moreover, such teachers dedicate their own time and energy to helping students with their problems. They know that the only thing that is predictable is unpredictability, meaning that there is no stable situation in which one can learn the most and the best, as a result of which they are aware of the fact that obstacles may arise while endeavoring to achieve goals and strategies should be taken into consideration to mitigate the suffering in such a situation; hence, they try to look on the bright side and view the problems as challenging through which knowledge can be boosted. Another highlighted point is that language itself is not enough to be taught; however, cultural differences should be taught in order to raise one’s knowledge. Optimism and TPC are so interrelated that they facilitate every step of the learning and teaching process because they help teachers see the problems as opportunities through success and as a result of which commitment can be taken. For example, students’ feedback is regarded as a positive factor for teachers’ well-being and success. Another example which can be taken into account is that such teachers see teamwork as a praiseworthy item through which knowledge can be shared and solutions can be found even though for some collaborative work is when one can brag about his achievements. Doing research for these teachers, additionally, is a great opportunity to increase their knowledge that is regarded as good for teaching.

It should be stressed that teacher commitment and academic optimism can not only be positively correlated, but they can also be negatively associated with each other in that optimism sometimes encourage people to be over positive about the situations, leading to not considering all the aspects to tackle a problem and not having enough insights to see both the positive and negative factors, facing a problem in educational contexts. It can be taken as an example, when a student just starts learning a language, he may have difficulty understanding the grammatical structures; however, as time passes, improvements can be seen. But if one could not enhance over a specific period of time, he might not be emotionally intelligent to perceive the grammatical structures even if his teacher urged him to do his best. Hence, it can be concluded that just because the teacher was optimistic about the learning process, it does not necessarily mean that the teacher can remain committed to teach such students and help them improve their language abilities since they may lose their interest to see any development in students with the passage of time. Even though no studies have so far been done to analyze the adverse effect of teacher optimism on their commitment, it can be regarded as important and avid teachers can put this hypothesis into practice.

The findings which have been gained gives credence to the following studies. Psychological capital can significantly predict teachers’ academic optimism and teachers’ job commitment. As it was proposed optimistic people are believed to always look on the bright side and it is what causes them to stay hopeful and motivated when facing difficulties, meaning that there is a positive correlation between TPC and AO (Gordon and Coscarelli, 1996; Seligman, 2002; Schueller and Seligman, 2008; Duckworth et al., 2009). According to Duckworth et al. (2009), it is believed that positivity or negativity is linked to the way people tackle their problems. It has been stressed in some studies that teachers with high levels of optimism are highly likely to teach more effectively because they can be risk-takers. Moreover, they grow a positive approach to their students and obstacles are regarded as new opportunities that can be dealt with. Therefore, this study is in congruence with the present study. However, since in the present study the emphasis was placed on psychological capital, it is a more comprehensive research. The same goes with a study conducted by Dong and Xu (2022) which focused their attention on EFL teachers’ optimism and commitment in their work engagement; hence, psychological capital has played no role in the aforementioned study. It was highlighted that the more optimistic and committed a teacher is, the more engaged he can be in his work. The current study is also aligned with a study carried out by Lu (2021) in which it was emphasized that EFL teachers’ optimism and their commitment make a contribution to students’ academic success. Even though both constructs were discussed considering students’ success, it had nothing to do with teachers who have a paramount role in educational contexts.

It has been emphasized by Schueller and Seligman (2008) that optimistic people are believed to always see the glass as half full and it is what causes them to keep hopeful and motivated in the face of difficulties. However, that study could be seen from a broader aspect, optimism can be related to two other important constructs. As it was confirmed in the current study teachers with higher psychological capital can be more optimistic and as a result of which they can be more committed.

Likewise, resilient teachers are believed to be more committed to their workplace and it is more probable to help their colleagues if they confront problems, to develop a friendly working ambience, to put up with inconveniencies without complaining, and to be more pleased with their jobs; therefore, they can manage the classes better (Witt, 1991). As a result, it is aligned with the findings of this study since it was found that there is a positive association between TPC and Teachers’ job commitment. Furthermore, the results are also in congruence with a study conducted by Snyder et al. (1991) since it has been shown that teachers with higher hopes are very likely to see students’ misbehaviors as the opportunities that help teachers to manage the class, utilizing new strategies. In other words, hopeful teachers have the capability to cope with the hindrances which do not allow them to achieve their goals in comparison to their counterparts with low levels of hope. Thus it is an emblem of a positive link between TPC and their commitment because the more committed a teacher feels, the more they make an effort to improve their working atmosphere that is relevant to both his PC and academic optimism.

## Conclusion and Implications

This research was carried out to examine the role of Chinese EFL teachers’ psychological capital in their job commitment and academic optimism. As the results of regression analyses revealed, Chinese EFL teachers’ psychological capital was found to be a significant antecedent of job commitment and academic optimism. Simply put, it was found that Chinese teachers’ job commitment and academic optimism can be notably improved by their psychological capital. That is, the higher the teachers’ psychological capital, the greater their job commitment and academic optimism. These results appear to be illuminating and insightful for teacher educators and EFL teachers. Given the value of teachers’ psychological capital in increasing their job commitment and academic optimism, teacher educators are expected to train teachers how to develop and maintain a positive psychological state. In this regard, teachers are also required to improve their psychological capital by regulating the negative emotions they typically experience in educational contexts.

In a modern world, considering the educational era, there are many stressors that slap energy and do not allow a person to be fully productive. Teachers are not exceptions though because their job puts them on the strain every now and then and wants them to be up-to-date with new materials or new teaching methods, therefore, it would be of utmost significance to find a way to still feel positive and as a result of which committed, facing difficulties. That is the reason why this study can be extremely important for both teachers and educational authorities. When teachers are aware of the situations in which they may feel pessimistic, they will learn how to keep their spirit up and not be influenced by negative experiences and issues. It is easier said than done to feel how pressurized a teacher may feel when under stress and it will not allow them to decide wisely and teach productively. Even if they are committed, they cannot be their best version and do the things as they are supposed to be done. Likewise, applying this study, educational authorities can provide teachers with the methods through which TPC can be raised in teachers and both their enthusiasm and commitment can be elevated have a positive impact on how they teach and what teaching methods and strategies they utilize.

There have been limitations associated with this study. Firstly, it is a quantitative study and open-ended questions are not asked which may find deeper truths about teachers; therefore, a longitudinal study that can be conducted over time seems necessary. Secondly, this research was done in China; hence, the findings might be different if the same research will be conducted in other regions since cultural diversity is a factor that should not be neglected. Thirdly, the sample size could be bigger than it already was; thus, attention should be paid to the point that the bigger a sample is, the more reliable it will be and future studies can be conducted considering this point. Finally, psychological capital is a newly-raised topic that can be scrutinized associated with other variables; consequently, avid researchers can carry out such studies.

## Data Availability Statement

The raw data supporting the conclusions of this article will be made available by the authors, without undue reservation.

## Ethics Statement

The studies involving human participants were reviewed and approved by Xi’an University of Posts and Telecommunications and Xi’an Jiaotong University Research Committee. The patients/participants provided their written informed consent to participate in this study.

## Author Contributions

LX designed this study and questionnaire and wrote up the research methodology, discussion, and conclusion. XZ analyzed the data and drafted the introduction and literature. Both authors have approved the final version before submission.

## Conflict of Interest

The authors declare that the research was conducted in the absence of any commercial or financial relationships that could be construed as a potential conflict of interest.

## Publisher’s Note

All claims expressed in this article are solely those of the authors and do not necessarily represent those of their affiliated organizations, or those of the publisher, the editors and the reviewers. Any product that may be evaluated in this article, or claim that may be made by its manufacturer, is not guaranteed or endorsed by the publisher.
